# Gut Microbiota and Cancer: From Pathogenesis to Therapy

**DOI:** 10.3390/cancers11010038

**Published:** 2019-01-03

**Authors:** Silvia Vivarelli, Rossella Salemi, Saverio Candido, Luca Falzone, Maria Santagati, Stefania Stefani, Francesco Torino, Giuseppe Luigi Banna, Giuseppe Tonini, Massimo Libra

**Affiliations:** 1 Department of Biomedical and Biotechnological Sciences, Oncologic, Clinic and General Pathology Section, University of Catania, 95123 Catania, Italy; silvia.vivarelli7@gmail.com (S.V.); rossellasalemi@alice.it (R.S.); scandido@unict.it (S.C.); luca.falzone@unict.it (L.F.); 2 Department of Biomedical and Biotechnological Sciences, Section of Microbiology, University of Catania, 95123 Catania, Italy; m.santagati@unict.it (M.S.); stefanis@unict.it (S.S.); 3 Department of Systems Medicine, Medical Oncology, Tor Vergata University of Rome, 00133 Rome, Italy; torino@med.uniroma2.it; 4 Division of Medical Oncology, Cannizzaro Hospital, 95126 Catania, Italy; gbanna@yahoo.com; 5 Department of Medical Oncology, University Campus Bio-Medico of Rome, 00128 Rome, Italy; G.Tonini@unicampus.it; 6 Research Center for Prevention, Diagnosis and Treatment of Cancer, University of Catania, 95123 Catania, Italy

**Keywords:** microbiota, microbiome, cancer, anti-cancer therapy, integrated therapy, inflammasomes, probiotics, *Lactobacillus rhamnosus GG*

## Abstract

Cancer is a multifactorial pathology and it represents the second leading cause of death worldwide. In the recent years, numerous studies highlighted the dual role of the gut microbiota in preserving host’s health. Gut resident bacteria are able to produce a number of metabolites and bioproducts necessary to protect host’s and gut’s homeostasis. Conversely, several microbiota subpopulations may expand during pathological dysbiosis and therefore produce high levels of toxins capable, in turn, to trigger both inflammation and tumorigenesis. Importantly, gut microbiota can interact with the host either modulating directly the gut epithelium or the immune system. Numerous gut populating bacteria, called probiotics, have been identified as protective against the genesis of tumors. Given their capability of preserving gut homeostasis, probiotics are currently tested to help to fight dysbiosis in cancer patients subjected to chemotherapy and radiotherapy. Most recently, three independent studies show that specific gut resident species may potentiate the positive outcome of anti-cancer immunotherapy. The highly significant studies, uncovering the tight association between gut microbiota and tumorigenesis, as well as gut microbiota and anti-cancer therapy, are here described. The role of the *Lactobacillus rhamnosus GG* (LGG), as the most studied probiotic model in cancer, is also reported. Overall, according to the findings here summarized, novel strategies integrating probiotics, such as LGG, with conventional anti-cancer therapies are strongly encouraged.

## 1. Introduction

Cancer is the second leading cause of death worldwide [1,2]. Cancer formation is the result of the stochastic intracellular accumulation of spontaneous mutations during DNA replication, combined with the environment exposure and lifestyle habits, both able to significantly influence cancer risk [3,4]. For example, the exposure to infectious agents, UV radiation and toxic substances, individual diet and lifestyle, strongly influences cancer rise, although the risk mainly depends on the dose, the duration and the combination of such injuries, coupled with the individual genetic background [5].

In the recent years, numerous evidences pointed towards the central role of commensal bacteria colonizing body surfaces as key determinants of health or pathologic conditions, including cancer [6]. Among the human symbiotic microbial populations, the gut microbiota is the most extensively studied and it deeply influences host’s homeostasis [7]. Gut microbiota is the name given to the heterogeneous population of commensal microorganisms, mainly bacteria, but also fungi, archaea and viruses, populating the intestinal tract, mostly the large intestine, and it can be considered as one factor to which we are persistently exposed, at high doses, throughout the entire lifespan [8]. Among a 1000 of different bacterial species within the gut microbiota, the highly represented ones are the *Firmicutes* and *Bacteroidetes* phyla [9]. The gut microbiota performs a number of vital functions, including production of vitamins, metabolization of dietary compounds, protection against the expansion and systemic infiltration of gut pathogens [10,11,12]. The gut microbial balance has a key role in the correct fulfilment of all these pivotal metabolic functions. Any imbalance in this delicate equilibrium may lead to an impaired microbiota, condition called dysbiosis, linked with several human pathologies, including cancer [13].

The gut microbiome, defined as the whole genome of the host’s gut microbiota, encodes 100-fold more genes than the human genome [14]. Within the last decade, the advent of metagenomics, combining next-generation sequencing (NGS) with computational analysis of the 16S rRNA amplicons, allowed to characterize both diversity and abundance of the gut microbiome. Progresses in metagenomics studies, together with the advancements in transcriptomics and metabolomics, allowed to describe the impact of individual bacterial species on host’s health [15,16]. That represents a step forward, from descriptive microbiome composition analyses, to functional studies, which are nowadays helping to understand the true impact of the microbiome architecture on human health [17,18]. However, this research is at its beginning, and all these growing number of associative and functional studies need to be further corroborated by results obtained from larger clinical studies [19].

Among all the pathologies linked with the gut microbiome, tumorigenesis is one of the mostly studied. The link has been found both with local gastro-intestinal cancers, as well as with other distal tumors [20]. Metabolomics and metagenomics studies highlighted the dual role of the gastro-intestinal microbiome in cancer prevention, tumorigenesis and anti-cancer therapy [21]. In fact, the gut microbiome can either be tumor-suppressive or oncogenic [22,23]. Although this link is studied since long ago, it is only partially characterized. In fact, all the current knowledge emphasizes the complexity and bidirectionality of the connection existing between microbiome and cancer. As a consequence, cancer development may alter the microbiome and, in turn, microbiome changes may affect cancer progression [24].

In this review, the up-to-date studies uncovering the tight link between gut microbiota and tumorigenesis will be described. Moreover, the importance of probiotics supplementation with anti-cancer therapy will be also discussed, including the key role and the future applications of a probiotic model studied in cancer, *Lactobacillus rhamnosus GG* (LGG), often used to treat cancer patients’ intestinal dysbiosis.

## 2. Gut Microbiota and Host’s Tumorigenesis

### 2.1. Gut Microbiota and Host Crosstalk

The gut is bidirectionally connected with the nervous system through the so-called “gut-brain axis” (GBA), which includes the central nervous system (CNS), the autonomic nervous system (ANS), the enteric nervous system (ENS), the hypothalamic pituitary adrenal (HPA) axis and the entero-endocrine system (EES). Hormones and neuro-hormones secreted at each of these listed GBA levels may modulate the gastro-intestinal digestive and metabolic activities, and vice-versa [25].

For that reason, the gut represents a complex interface between the gastro-intestinal resident microbiota and the human body. There is a bidirectional crosstalk between gut resident microbes and host’s GBA, in which the gut functions as the communication gatekeeper [26]. In particular, it is known that host’s hormones and neuro-hormones are able to modify the gut microbiome composition, as during stress response [27]. Importantly, the gastro-intestinal entero-endocrine cells secrete over 30 different peptide hormones, involved in several functions, such as gastro-intestinal motility, digestive functions and neuromodulation [28]. A number of these hormones can be sensed by the gut bacteria, as in the case of leptin and ghrelin, which, in turn, finely tune the gut microbiota composition in rodents [29,30].

In the same way, the gut microbial population secrete active molecules which can be sensed by the gut cells and whose effects are then transduced to the GBA [27]. In fact, it has been widely studied that the gut microbiota may produce or transform molecules affecting several aspects of human health, including: host’s metabolism modulation, gut barrier integrity maintenance, xenobiotics and drug metabolism, protection against gastro-intestinal pathogens and host’s immune system modulation [31,32,33,34].

With regards to the host’s metabolism, it is known that certain commensal bacteria produce essential micronutrients, such as vitamin K and various components of vitamin B. Also, members of the *Bacterioides* family are able to synthetize the anti-diabetic compound linoleic acid, to catabolize secondary bile acids and to break down phenolic compounds. Moreover, a number of gut commensals can modify small aminoacids into signaling molecules, as for example histidine to histamine or glutamate to γ-aminobutyric acid (GABA) [35].

Additionally, it has been reported that gut resident commensals can produce hormone-like metabolites, such as the short chain fatty acids (SCFAs), as a consequence of the bacterial fermentation of dietary fibers in the large intestine [36]. The nature of the SCFAs synthetized depends on both diet and microbiota specific composition. The SCFAs, once produced by the gut bacteria, are transported through the bloodstream, and used by the liver as the main source of energy. Importantly, SCFAs have a role in controlling the glucose and the lipidic metabolism, by affecting the intestine hormone peptide secretion, including Peptide YY (PYY) and Glucagon-like peptide 1 (GLP-1) [37].

### 2.2. Gut Microbiota as a Tumor-Suppressor

Given the above illustrated complex crosstalk occurring between the host and its gut microbiota, it is not surprising that the gut microbial population may affect pathological processes, such as cancer genesis and development, either in a positive or in a negative way, depending on its own composition. Remarkably, a number of microbes-derived molecules show an anti-tumor activity.

In particular, microbial-derived SCFAs may have an anti-cancer effect. For example, gut bacterial butyrate and propionate are able to inhibit host’s tumor cells histone deacetylases with a general anti-cancer effect. Such mechanism is the cause of the anti-tumoral in vitro and in vivo effect of butyrate observed in both colorectal cancer (CRC) and lymphoma [38,39]. Some of the probiotics’ derived molecules and metabolites are able to modulate host’s immune system, thereby triggering an indirect immune-mediated response against tumor development. For example, the widely studied bacterial lipopolysaccharide (LPS), a major component of the outer membrane in gram-negative bacteria, activates the host’s cell surface receptor toll-like receptor 4 (TLR4), belonging to the family of pattern recognition receptors (PRRs), thus activating immune T cell-mediated response against cancer cells [40]. In the same way, the monophosphoryl lipid A (MPL) from *Salmonella enterica* has been currently used as adjuvant in the vaccine formulation used against anti-cervical carcinoma [41]. Moreover, bacterial derived pyridoxine, a group B vitamin, can stimulate host’s antitumoral immunosurveillance [42].

**Figure 1:**
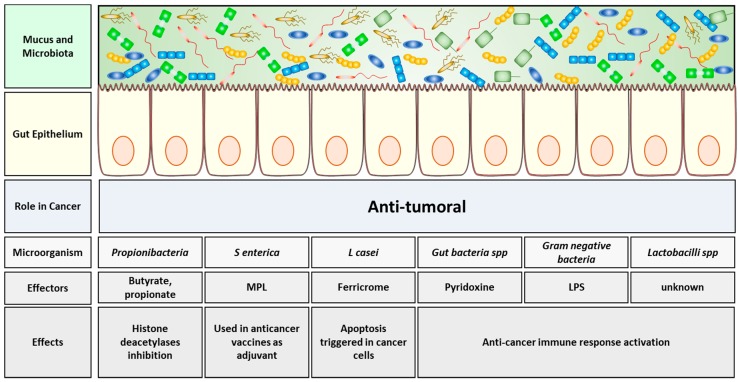
Anti-tumoral effects of the gut microbiota. Probiotics and other gut resident bacteria are able to secrete molecules, capable, in turn, to fight tumor growth and prevent tumorigenesis through several mechanisms. Schematic of the intestinal layers, from top to bottom: mucus and microbiota, gut epithelium. Into the grey boxes are illustrated, from top to bottom, the microorganism species implicated in the anti-cancer process, the molecules produced and the corresponding effects induced within the host. Abbreviations: MPL, monophosphoryl lipid A; LPS, lipopolysaccharide.

Several commensal bacteria play a probiotic role thanks to their capability to confer health benefits, either protecting against gut dysbiosis or enhancing host’s immune defense mechanisms [22,24]. The administration of such probiotics, as for example Mutaflor (*Escherichia coli* Nissle 1917) combined with the intestinal antibiotic rifaximin, demonstrated a clear anti-inflammatory activity, enhancing the anti-inflammatory effect of rifaximin in a rat model of inflammatory bowel disease [43]. Additionally, many probiotics have shown a potential antineoplastic activity. For example, probiotics or probiotics-derived metabolites administered to mice can, in turn, to inhibit tumor growth. One good example is ferricrome metabolite secreted from *Lactobacillus casei*, able to trigger apoptosis in tumor cells via JNK pathway direct activation [44]. It has been also reported in several studies that Lactobacilli may stimulate host’s immune cells such as NK cells or dendritic cells (DC) or TH1 response, which, in turn, leads to the elimination of cancerous or precancerous cells, although the exact bacterial bioproduct mediating such stimulatory effect still needs to be identified [45,46,47,48]. A summary of the gut microbiome anti-cancer functions is illustrated in Figure 1.

### 2.3. Gut Microbiota as a Tumor-Promoter

Gut dysbiosis and the consequent development of pathogenic populations within the gut microbiota, may contribute to a wide variety of pathologies, even in sites distant from the gut, ranging from bowel inflammation, to neurodegenerative diseases (including Parkinson’s disease) and cancer [43,49,50]. 

Regarding cancer, within a dysbiotic gut, certain bacterial pathogens can negatively affect either the host’s metabolism or the host’s gut and immune system functionalities, thereby triggering tumor growth [51]. Importantly, gastro-intestinal dysbiosis has been linked with both local and distant tumors [52]. Microbial pathogens are known to drive the 20% of tumorigenesis and a larger number of malignancies are associated with microbial commensal imbalance, or dysbiosis [53]. In line with that, many preclinical studies performed using germ-free mice models demonstrated how the gut microbiome is able to deeply affect cancer genesis and progression through different mechanisms [33,54,55].

*Helicobacter pylori* produced protein CagA was the first bacterial protein shown to be involved in human cancer [56]. Although only *Helicobacter pylori* is included among class I carcinogens by the World Health Organization (WHO) [57], several studies performed in cell culture and animal models, assessed the ability of additional microbiota populations to affect host’s DNA replication and integrity [58,59,60]. In fact, during pathogenic infections, when the gut microbiome is affected by dysbiosis, bacterial pathogens can expand and release a large amount of toxins which, in turn, induce host’s DNA breaks, thus contributing to genomic instability, tumor initiation and progression in those predisposed cells [61,62,63]. This is the case of colibactin and cytolethal distending toxin (CDT) both produced by *Escherichia coli* and displaying a DNAse activity. Once released in the proximity of the gastrointestinal epithelium, the toxins generate DNA double-strand breaks within the host’s epithelial cells, thus promoting a transient cell cycle arrest, allowing for genomic mutations to arise, and finally leading to tumor formation [64]. Gut pathogenic bacteria can also interfere with DNA damage response and repair pathways, as in the case of *Shigella flexneri*, inducing host’s cells p53 degradation via the secretion of its enzymes inositol phosphate phosphatase D (IpgD) and cysteine protease-like virulence gene A (VirA), therefore increasing the probability of introducing mutations during the DNA damage response in infected cells [65]. In the same way, the product of the cytotoxin associated gene A (CagA) from *Helicobacter pylori*, induces the proteasome-mediated degradation of p53 in gastric epithelial cells, by interfering with the host’s AKT pathway, thus promoting the rise of gastric cancer [66].

Moreover, gut bacteria can modulate several host’s cellular proliferative and pro-survival pathways, therefore contributing to cancer. For example, *Helicobacter pylori* derived CagA protein, *Fusobacterium nucleatum* effector adhesin A (FadA) and *Bacteroides fragilis* metalloproteinase toxin (MP toxin) are all capable to interact (directly or indirectly) with the host’s epithelial E-cadherin, thus disrupting the intercellular junctions and activating β-catenin signaling. This, in turn, triggers cell proliferation and the potential cancerogenic transformation of those affected host’s cells [67,68,69]. In the same direction, the *Salmonella enterica* effector avirulence protein A (AvrA) is able to translocate into host’s cells and activate β-catenin via its intrinsic de-ubiquitinase activity [70].

As for the β-catenin signaling, other virulence factors released in the extracellular gut milieu during a pathogenic infection, can potentially induce cancer transformation when infecting pre-transformed cells, through the activation of other pro-survival intrinsic host’s cellular pathways, such as MAPK and AKT, as for CagA from *Helicobacter pylori*, controlling the hosts MAPK pathway or AvrA from *Salmonella enterica*, triggering both MAPK and AKT pathways [71,72]. In particular, CagA from *Helicobacter pylori* can bind many host’s proteins intracellularly, including the protein tyrosine phosphatase SHP-2. CagA-SHP-2 complex formation deregulates the phosphatase activity of SHP-2, which, in turn, promotes Ras/MAPK signaling activation [73].

Additionally, pathogenic bacteria may indirectly affect host’s tumorigenesis. Different mechanisms can mediate this effect. One is the generation of oxidative stress, leading to cell autonomous genomic mutations [74,75]. Another one consists either in the enhancement of the inflammation or the inhibition of the host’s immune response, thus helping the tumor immune-escape [76]. For example, *Helicobacter pylori* or *Bacteroides fragilis* are both able to activate the host’s spermine oxidase, which, in turn, generates hydrogen peroxide and reactive oxygen species (ROS)-induced accumulation of DNA damage [77,78]. *Enterococcus faecalis* produces extracellular superoxide and derivative oxygen species is capable to diffuse into host’s cells. In turn, the increase in the oxidative milieu enhances the possibility of host’s cellular DNA mutations [79].

Moreover, relevant bacteria can stimulate cancer formation by blocking immune-effectors that normally inhibit tumorigenesis. For example, *Fusobacterium nucleatum* inhibits for its own advantage host’s Natural Killer (NK) cells, in order to recruit at the site of the infection myeloid suppressor cells, therefore indirectly helping cancer genesis. Such mechanism is mediated by the bacterial virulence factor Fap2, able to bind and block the NK inhibitory receptor TGIT, thus arresting the NK-mediated tumor cell attack [80].

Finally, certain microbiota species may interfere with host’s hormones metabolism. In fact, it has been widely studied the link between bacterial secretion of the β-glucuronidase enzymes and the increased bioavailability of the host’s estrogen hormones (both originating from hepatic catabolism and phytoestrogens). When gut dysbiosis is coupled with an increase in the β-glucuronidase-secreting bacteria, such as *Clostridium leptum* and *Clostridium coccoides*, the enzyme deconjugates liver-catabolized and plant-derived estrogens, enabling them to bind and activate the estrogen receptors expressed by target cells [81]. Estrogen receptors activation promotes cell proliferation in tissues responding to estrogens, as breast and endometrium [82]. Accordingly, this augmented intake of estrogen hormones is linked with an increased risk of developing breast cancer, supporting the finding that the gut microbiota composition of women with breast cancer differs from that from healthy controls, and suggesting that several gut bacteria, which could be over-expressed during dysbiosis, may be linked with breast cancer development [83].

**Figure 2:**
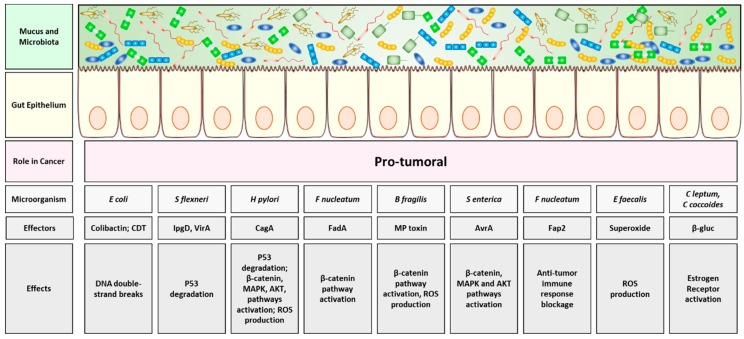
Pro-tumoral effects of the gut microbiota. Bacteria prominent during gut dysbiosis can secrete toxins able to interfere with host cell growth, finally predisposing the host organism to cancer development. Schematic of the intestinal layers, from top to bottom: mucus and microbiota, gut epithelium. Into the grey boxes are illustrated, from top to bottom, the microorganism species implicated in the pro-cancer process, the molecules produced and the corresponding effects induced within the host. Abbreviations: ROS, Reactive Oxygen Species; CTD, cytolethal distending toxin; IpgD, inositol phosphate phosphatase D; VirA, virulence gene A; CagA, cytotoxin associated gene A; FadA, Fusobacterium effector adhesin A; MP Toxin, metalloproteinase toxin; AvrA, avirulence protein A; β-gluc, β-glucuronidase.

Although there are notable examples of pathogenic microbiota capable of promoting oncogenesis through the modulation oncogenic host’s cell pathways or by interfering either with the host’s hormonal or the host’s immune system, no strong bacterial oncogenic driver has been identified yet. In particular, it is difficult to clearly determine whether microbiota changes might affect cancer genesis or the contrary [84]. Additionally, changes in the host’s lifestyle, diet and immune system are among the factors which deeply influence the microbiota composition and activity [85]. Moreover, the very same anti-cancer treatment might shape the patient’s microbiome and, at the same time, host’s specific microbiome can deeply affect patient’s response to therapy [19]. A summary of the gut bacteria pro-tumoral functions is schematized in Figure 2.

## 3. *Lactobacillus rhamnosus GG*: A Probiotic Model in Cancer

Due to its anti-inflammatory properties, the probiotic archetype *Lactobacillus rhamnosus GG* (LGG) is one of the most studied and well characterized among probiotics. The probiotics, including *Lactobacilli*, are studied as supportive treatment for chemotherapy-associated gastrointestinal toxicity, thanks to their ability to restore gut microbial balance, as further described in Section 5 [86]. Among these probiotic species, LGG is one of the first studied specifically in oncology [87]. LGG is a gut resident bacterium known to have several anti-inflammatory effects within the intestinal microenvironment [88,89,90]. In animal models, LGG administered with food attenuates 5-FU-mediated as well as radiation-mediated gut epithelial injury, therefore helping the preservation of the gut microbiota balance and the intestinal epithelial barrier functionality [91,92,93].

Several potential benefits of LGG administration to cancer patients have been foregrounded since long time, by in vitro, in vivo and clinical studies, as recently reviewed by Banna et al. [94]. In line with these studies, a number of ongoing clinical trials are currently focused on establishing the role of LGG administration in preventing or ameliorating the toxic effects of anti-cancer therapies (Table 1). Additionally, two clinical trials have been designed by our research team and approved by the local ethical committees; while the ClinicalTrials.gov identifiers (NCT numbers) are on their way to be assigned. The two studies are entitled respectively: “Maintenance of normal gastro-intestinal function with dietary supplement containing *Lactobacillus rhamnosus GG* in cancer patients treated with cytotoxic chemotherapy and/or targeted therapy” and “Maintenance of normal gastro-intestinal function with dietary supplement containing *Lactobacillus rhamnosus GG* in patients treated with abdominal or pelvic radiotherapy”. The two trials are aimed to assess the efficacy of LGG daily oral administration in the maintenance of normal gastro-intestinal functions in cancer patients treated either with chemotherapy and/or targeted therapy or abdominal/pelvic radiotherapy. In addition, the effects of such dietary supplementation on both the patients’ intestinal microbiome composition and also in their circulating microRNAs pattern will be further evaluated.

Given the beneficial role of LGG in ameliorating the anti-cancer therapy-related side effects, many groups are investigating also the potential role that LGG might have in the direct modulation of cancer development. In particular, it has been observed that LGG exerts its effect either directly on cancer cells or indirectly through the modulation of the immune system, both in vitro and in vivo.

Firstly, LGG is capable of counteracting cancer growth. It has been demonstrated within several in vitro tumor models (including colorectal, ovary, breast, cervical, hepatic and oral squamous) that LGG is able to exert either anti-proliferative effects or anti-metastatic effects [102,103,104,105,106]. This could be mediated through the direct modulation of several host’s proliferation pathways, such as mTOR or WNT [107]. In addition, LGG prevents polyps’ formation in a colorectal APC/min mouse cancer model, and reduces colitis-associated cancer in mice [108,109].

Secondly, LGG can influence host’s immune system, therefore helping the host to eliminate newly developing cancer cells. In fact, treatment with LGG in a rat dimethyl hydrazine-induced colon cancer model, is able to reduce the tumor mass through the modulation of the commensal gut microbiome and the downregulation of pro-inflammatory molecules produced by both gastrointestinal cells and gut-resident immune cells [110]. Additionally, host’s DC exposed to LGG induce TH1 immune cells polarization and, in turn, antitumor immune-response potentiation [111].

LGG triggers the immune response also within the normal not transformed gut epithelium, thus protecting towards inflammation, which can support the formation of a cancer-favorable milieu [112]. On this line, it has been recently observed how LGG attenuates NLRP6-mediated inflammasomes activation in the intestine [113]. LGG administration can also change gene expression in intestinal porcine epithelial cells and intestine myo-fibroblasts towards an anti-inflammatory profile [114,115]. A complete list of the latest in vitro and in vivo studies untangling the complex role of LGG in cancer development is reported in Table 2.

Altogether, the currently ongoing clinical studies (dissecting the beneficial effects of LGG administration during anti-cancer therapy), coupled with the in vitro and in vivo studies (supporting LGG as a direct cancer modulator), make LGG a suitable candidate to be further characterized as possible adjuvant in integrated anti-cancer therapies.

## 4. Gut Microbiota and the Inflammasomes

In addition to the role of gatekeeper with the GBA, the gut represents also the interface between the microbiome and the host’s immune system. The gut microbial population plays a key role in training, functioning and stimulating the host immune system, with the final effect of developing tolerance towards beneficial microbiota and eliciting immune response against gut pathogens [76].

Within the Section 2 and Section 3 of this review, how the gut microbiome is able to deeply influence the host’s immune and inflammatory responses, with either protective or detrimental effects on the oncogenesis, depending on the nature of the bacteria and the host’s immune cells involved, has been described. Additionally, gut microbiome interacts both with immune cells and gut cells through the activation of the so-called inflammasomes. Inflammasomes are multiprotein intracellular complexes expressed by both immune cells and epithelial cells. They can detect pathogenic and non-pathogenic microorganisms-derived molecules, as well as sterile stressors molecules, via a subset of cytoplasmic pattern recognition receptors (PRRs), called NOD-like Receptors (NLRs) [116].

Given the ability of such multiprotein complexes to sense both microbial and endogenous stimuli, inflammasomes are regarded as the guardians of cellular and tissue integrity. Upon disruption of host’s homeostasis and activation of inflammasomes sensors, a strong inflammatory response is activated. In detail, the inflammasomes complex mediates the activation of caspase-1 which, in turn, triggers the secretion of the proinflammatory cytokines interleukin-1β (IL-1β) and interleukin-18 (IL-18) as well as pyroptosis, a form of programmed cell death [117]. Dysregulation of inflammasomes results in a variety of diseases, ranging from autoimmunity to cancer [118]. Importantly, among all the stimuli, inflammasomes are also capable of sensing host-microbiota interactions, thus taking an active role in response to commensal and pathogenic bacteria [116].

The role of the inflammasomes in tumorigenesis is controversial. In fact, the inflammasomes have been shown to be either tumor-promoting or tumor-suppressive, depending on the nature of both the tumor and its microenvironment. The exact outcome of the inflammasomes activation depends on multiple factors, including its expression pattern and effector molecules, together with the tumor nature and stage. Importantly, the gut microbiome may also influence the outcome of the specific inflammasomes activation [119]. Currently, we have very limited knowledge on the mechanisms responsible for inflammasomes activation during tumor development. Multiple studies using different mice deficient for inflammasomes components (including NLRP3, NLRP1, NLRP6, NLRC4 and Caspase-1) found that the inflammasomes protect mice from colitis-associated CRC [120,121,122,123,124]. Importantly, the inflammasomes effector IL-18, but not IL-1β, plays a pivotal role in suppressing colitis. In fact, IL-18 KO or IL-18R KO mice are also highly sensitive to colitis-associated CRC [124,125]. All these studies highlight the importance of inflammasomes-dependent IL-18 production in suppressing CRC. On the contrary, inflammasomes activation results detrimental to the development of lung, skin, breast and pancreatic cancer. In those cases, IL-1β is the key player, acting as pro-inflammatory, tumor-promoting trigger [126,127,128,129,130].

The commensal microbiota and their bioproducts are sensed by epithelial cells and innate immune cells via innate receptors, including the inflammasomal NLRs. In particular, inflammasomes-mediated IL-18 is critical for intestinal tissue remodeling and gastro-intestinal barrier maintenance. Commensal bacteria and their bioproducts induce inflammasomes activation and IL-18 production in the gut, which, in turn, prevents intestinal barrier disruption and dysbiosis [121,124,131,132,133]. Deficiency in inflammasomes components leads to reduced production of IL-18, resulting in an intestinal barrier impairment. Such damage causes larger commensal bacteria penetration, and increased inflammation, which may finally trigger tumorigenesis. Dysbiosis has been observed in mice deficient for inflammasomes components, including NLRP6, ASC, caspase-1, and IL-18 [131,132,133,134]. Importantly, it has been proposed that inflammasomal NLRP6 is required for the commensal bacteria homeostasis. NLRP6 KO mice show dysbiosis and increased incidence of inflammation-associated CRC [135,136,137]. Inflammasomes and IL-18 are protective in inflammation-induced CRC. Further studies are needed to assess whether inflammasomes and/or IL-18 inhibit CRC development in genetic CRC models (such as the APC/min mice), as well as in human CRC. Relevantly, *Lactobacilli* are able to activate the inflammasomes in human primary macrophages, as well as in primary mammalian gut epithelial cells, as defense mechanism against viral infection or epithelial injury [138].

Therefore, inflammasomes represent a double-edged sword in tumorigenesis and the gut microbiome may influence the outcome of the specific inflammasome activation during tumorigenesis. In CRC inflammasomes activation has a protective role, on the contrary in breast and skin cancers their triggering leads to detrimental outcomes [119]. Regarding the correlation between inflammasomes, microbiome and cancer, NRPL6 plays a key role in colorectal carcinogenesis and, in particular, NLRP6 regulates susceptibility to intestinal inflammation through its microbiome-modulatory activity [139].

## 5. Gut Microbiota and Anti-Cancer Therapy

Anti-cancer therapies are designed with the final goal of being effective in the eradication of the targeted malignancy. Because almost every available anti-cancer treatment is toxic also towards normal cells, their use may be coupled with side effects, some of which can compromise the overall survival of the patients [140].

Additionally, tumors are intrinsically complex: as they attempt to accumulate mutations, cancers evolve and adapt to the hosting organism [141]. In fact, cancers derive from the stochastic acquisition of driver mutations within genes involved in key processes, including DNA duplication, DNA repair, oxidative stress response. Such accumulation finally allows the transformation of a normal cell into a malignant one [141]. Both the initiation and the progression of a tumor may be viewed as a blended impairment of such fundamental cellular processes, meaning that from one original cancer cell might derive a molecularly varied bulk tumor made of multiple clones of cancer cells, each one displaying a differential intrinsic sensitivity to the anti-cancer therapies [142]. This heterogeneity derives from intrinsic tumor cellular genomic instability, ranging from microsatellite instability (due to impairments of the DNA mismatch repair system) to chromosomal instability (arising from segregation errors during cell mitosis) [143,144]. On top of that, such genetic mechanisms might be coupled with epigenetics, transcriptional and post-transcriptional intracellular changes, finally leading to a growing tumor complexity, through time and space [145].

Importantly, this intra-tumoral variety is tightly linked with the development of the resistance to therapy, considered the first cause of failure of the available anti-cancer treatments, as well as subsequent tumor relapses [146]. To fight such resistance, integrated therapies and personalized approaches, based on the specific genetic features of the malignancy, are in continuous progress [146].

Developing malignant cells not only are subjected to their intrinsic heterogeneity, additionally they are recognized and eliminated by the host’s immune system [147]. On their side, tumor cells, thanks to their genetic instability, constantly evolve novel strategies to escape from such immunosurveillance and expand within the host [147]. Along with chemotherapy and radiotherapy, a novel anti-cancer approach is considered the so-called targeted immunotherapy, bearing the dual role of both boosting the host anti-tumor immune response, and, at the same time, helping to hit cancer resistance and recurrence mechanisms [148,149].

**Figure 3:**
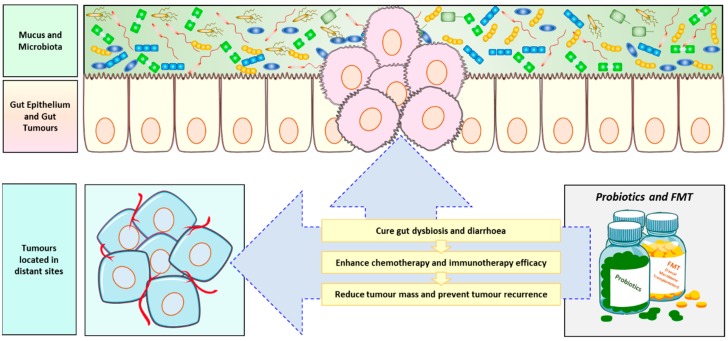
Role of probiotics in anti-cancer therapy. Probiotics and Fecal Microbiome Transplantation (FMT) are currently studied as anti-cancer adjuvants to fight dysbiosis following anti-cancer therapy, to increase chemotherapy and immunotherapy efficacy and to both reduce tumor mass and prevent tumor recurrence.

Modulating gut microbiome may deeply influence the outcome of anti-cancer therapies. In fact, radiotherapy, chemotherapy and immunotherapy treatments can all modify patients’ microbiome and, at the same time, microbiome composition can deeply affect patients’ response to such therapies [150]. It is therefore fundamental to identify which are the factors able to influence the gut microbiome and, in turn, to find novel strategies to manipulate the gut microbiome, with the main goal of finally improving patients’ therapeutic outcome. Specifically, interventions on microbiome may be pivotal to ameliorate anti-cancer therapy-related toxicity, as well as to improve anti-cancer therapy efficacy [151,152]. The emerging role of the gut microbiome in cancer therapy is summarized in Figure 3.

Back in 1890 two heat-inactivated microorganisms (*Streptococci*) were injected intratumorally for the very first time in humans as an attempt to cure cancer [153,154]. Furthermore, several decades later, *Mycobacterium bovis* was successfully injected into bladder in patients, following the resection of a bladder tumor. It has been observed that the bacteria, by inducing a local immune response, helped to reduce the relapse of the tumor [155]. Moreover, it has been discovered how oral administration of *Lactobacillus casei* decreased superficial bladder cancer recurrence [156]. The mechanism behind involves the direct bacterial stimulation of host’s NK cells and macrophages, in turn responsible of a strong antitumoral immune response [157].

These observations paved the way for many published, as well as ongoing, clinical trials, based on the usage of gut bacterial attenuated strains in anti-cancer therapy. These trials are shedding light on the key role of such bacteria on triggering anti-tumor immune response [158]. For example, it has been observed that the intradermal injection of *Mycobacterium obuense* in melanoma and in pancreatic ductal carcinoma activates antitumoral immune response, acting on host’s antigen presenting cells (APCs) and cytotoxic T cells [159,160]. Additional clinical trials further revealed how attenuated bacteria injected directly into the tumor mass are able to both stimulate anti-tumoral immune response and also have a direct cytotoxic effect on the tumor cells, because their capability of colonizing tumors, as observed in several different refractory solid tumor studies, towards the administration of attenuated and/or genetically modified *Salmonella typhimurium* [161,162,163]. Although these results are encouraging, many clinical trials are currently ongoing in order to ameliorate the patients’ clinical outcomes, given bacteria-associated toxicity, mainly correlated to their long half-life [163].

### 5.1. Modulation of Gut Microbiota to Enhance Chemotherapy and Immunotherapy Efficacy

The microbiota, when affected by dysbiosis, can deeply influence both cancer pathogenesis (as reported in Section 2) and its therapeutic outcome. In particular, the regulation of such therapeutic outcome is tightly connected with the ability of the gut microbiota to metabolize anti-tumoral compounds, as well as to modulate host’s immune response and inflammation pathways [164]. These two effects combined together may explain the strong involvement of the patient’s microbiome composition in affecting the efficacy of both chemotherapy and immunotherapy [19].

With regard to chemotherapy, it has been reported how tumor-bearing mice, either germ free or having their gut microbiota depleted after antibiotics therapy, do not respond to oxaliplatin drug treatment. The explanation is that commensal microbiome members within the gut of the mice might produce TLR agonists, thus promoting the rise of an oxidative stress milieu and tumor cell death. As a direct consequence, without a healthy gut microbiota there is a decreased microbiota-dependent ROS production, thus a less effective chemotherapeutic response [165]. Consistently, mice bearing lung tumors treated with cisplatin coupled with antibiotics, survive less and develop bigger tumors. If cisplatin is combined with probiotics, such as *Lactobacilli*, mice show an improved response to therapy. The mechanism involves the induction of pro-apoptotic genes within the tumor mass and the enhancement of host’s immune response [166].

Another widely used anti-cancer molecule, cyclophosphamide, coupled with oral bacterial administration (*Lactobacillus johonsoni* and *Enterococcus hirae*), leads to the conversion of T cells from naïve to pro-inflammatory T helper 17 (TH17), with the final effect of improving cyclophosphamide efficacy in tumor-bearing mice [167,168].

With reference to immunotherapy, the administration of CpG oligodeoxynucleotides, synthetic molecules mimicking bacterial DNA, strongly stimulate the immune system, therefore showing anti-tumor activity in several cancers [169]. Along this line, the intra-tumoral injection of CpG oligodeoxynucleotides administered together with an anti-interleukin-10 receptor (IL-10R) antibody, induce TNF production from tumor infiltrating myeloid cells and, in turn, reduce the growth several types of tumors in mice [165]. Moreover, the administration of a specific bacteria, *Alistipes shahii*, to antibiotic-treated tumor bearing mice, restores TNF production with a notable improvement in the therapeutic outcome [165].

Given the multiplicity of effects that gut microbiome may play on the host’s immune system, it is not surprising that emerging studies strongly linked the patients’ microbiome composition with the intrinsic efficacy of immune checkpoint inhibitors-based immunotherapy, in the treatments of different solid tumors [170,171,172]. Immune checkpoint inhibition consists in the administration of therapeutic agents able to block the immune-inhibitory pathway, thus modulating T cell activation against tumor target cells. The currently marketed checkpoint inhibitors are in monoclonal antibodies targeting cytotoxic T lymphocyte-associated protein 4 (CTLA4) or the programmed death 1 (PD1) located on T cell surfaces, or its ligand, programmed death ligand 1 (PD-L1), expressed by the APCs [173]. While CTLA-4 regulates T cells proliferation early in the immune response within the lymph nodes, PD-1 suppresses T-cell activation later, within the body periphery [174].

A few years ago, two studies suggested the potential involvement of the gut microbiome in modulating the efficacy of such anti CTLA4 and anti-PD1 based therapies [175,176]. Vetizou et al. demonstrated that the efficacy of anti-CTLA4 antibodies in reducing sarcoma tumor growth in mice is significantly increased when the gut microbiome is enriched in *Bacteroides fragilis* and *Burkholderia cepacia* [175]. On the same line, Sivan et al., found that the efficacy of PD-L1 targeting-antibody in the cure of melanoma in mice is improved in the presence of gut microbiome enriched in *Bifidobacterium* species [176]. In fact, they demonstrated that oral administration of a cocktail of *Bifidobacterium* species combined with the anti-PD-L1 antibody, specifically boosts T cell response and blocks the melanoma growth [176].

Multiple translational studies, published in 2018, further support the pivotal role of the gut microbiome in modulating the response to immune checkpoint blockade [170,171,172]. In particular, Routy et al. [170] found that melanoma patients treated with antibiotics along with the anti-PD1/anti-PD-L1 immunotherapy had a lower survival rate. Importantly, metagenomics analysis of patients’ fecal gut microbiome showed a difference in the composition of their gut microbiome. Anti-PD1 responders were enriched in two phyla (*Akkermansia* and *Alistipes*). Performing FMT from patients to germ free mice, the authors found that *Akkermansia muciniphila* (alone or in combination with *Enterococcus hirae*) was able to increase intra-tumoral cytotoxic T cell infiltrates, thus increasing the PD-1 blockade response in mice [170]. In parallel, Gopalakrishnan et al. [171] demonstrated through metagenomics analysis of melanoma patients’ fecal samples that the anti-PD1 responders’ microbiome was different in composition compared with that of non-responders. The authors observed in patient’s gut microbiome an increase in the abundance of *Clostridiales*, *Ruminococcaceae* and *Faecalibacteriae*. Functional studies performed with FMT in germ free mice further demonstrated how treating mice with the identified bacteria, along with the anti-PD1 therapy, enhanced the anti-cancer effects and reduces the melanoma growth [171]. Along the same line, Matson et al. [172] performed a metagenomics characterization of stools samples from melanoma patients treated with immune checkpoint inhibitors, further corroborating the finding that responders showed a different microbiome compared to those not responding to therapy. They identified and functionally proven in vivo the importance of *Bifidobacterium longum*, *Enterococcus faecium* and *Collinsella aerofaciens* in ameliorating anti-PD-L1 efficacy [172].

Although the immune checkpoint inhibitors have success in treating various malignancies, there is still a significant number of patients that can use such therapy only for a limited amount of time, given the occurrence of strong toxic side effects, including gut inflammation, due to the subsequent immune-dysregulation (i.e., autoimmunity) [177]. In animals, oral gavage of *Bacterioides fragilis* and *Burkholderia cepacia* demonstrated an amelioration of such immunotherapy-associated toxic side effects [175]. In line with this observation, it has been seen that patients treated with anti-CTLA4 antibody, toxic side effects are mediated by an increased abundance of *Firmicutes*, such as *Faecalisbacterium*, and a decreased abundance of *Bacterioides* [178,179]. Altogether, these data provide a strong evidence of the role of gut microbiota composition in modulating the effect of both immunotherapy response and toxicity.

Even if the last decade witnessed massive advances in unveiling the role of gut microbiome in cancer and other diseases, there are still many obstacles for translating basic microbiome research into therapeutic applications. Among the gut bacteria can develop potential pathogens and that could limit, or at least slow down, the translation of the in vitro and in vivo results to the clinic. In light of the novel studies reported above, any antibacterial therapy altering the intestinal equilibrium, during anti-cancer therapy, needs to be carefully evaluated. In fact, the heterogenous patients’ microbiome can either be detrimental or beneficial to tumor progression and therapy, depending on its composition and prevailing species. As further discussed below, looking at the effects of probiotics treatments in anti-cancer therapy, it might be necessary in the future to pursue a personalized approach, based on the specific patient’s microbiome composition.

### 5.2. Use of Probiotics in Oncology

As discussed above, chemotherapy, targeted therapy, immunotherapy and radiotherapy represent the pillars of the currently available anti-cancer treatments. Such treatments may cause diverse and even drastic side effects in patients [180,181,182,183,184,185,186]. Several preclinical studies and clinical trials share the common goal of evaluating the overall efficacy of probiotics in decreasing the risk and the severity of such anti-cancer treatments related-toxicity, mainly diarrhea and mucositis [187,188]. In fact, the aim of administering probiotics to cancer patients, principally *Lactobacilli*, is to re-populate the compromised patients’ gut microbiota, thus re-establishing the levels and functionality of the commensal bacteria, depleted after the treatments [189]. Although probiotics are generally regarded as safe, the main concerns of administering them to immunocompromised cancer patients are both the potential risk of opportunistic infection development and the transfer of antibiotics resistance [190,191]. Despite of that, probiotics administration in multiple trials has shown beneficial effects on ameliorating diarrhea and other gut-related damages following anti-cancer therapy, thus re-establishing a healthy intestinal microbiota composition [192]. Moreover, within the Multinational Association of Supportive Care in Cancer and International Society of Oral Oncology (MASCC/ISOO) and European Society of Medical Oncology (ESMO) Clinical Practice Guidelines for Gastrointestinal Mucositis, probiotics containing *Lactobacillus* species are suggested be used to prevent diarrhea in patients receiving chemotherapy and/or radiation therapy for a pelvic malignancy (Level of evidence III) [193,194].

Given that a growing body of studies corroborated the fundamental role of microbiome in cancer, many clinical studies are currently ongoing with the common aim of investigating the therapeutic potential of manipulating gut microbiota in cancer patients. Results from early clinical trials are promising. In 2010, it was assessed for the first time the interaction between probiotic administration, variation of gut microbiota composition, and regulation of intestinal immune-functions in cancer patients undergoing colorectal resection [95]. A mixture of two probiotic species *Bifidobacterium longum* (*BB536*) and *Lactobacillus johnsonii* (*La1*) was administered to the patients in the double-blind study, finding that one of that, *La1*, was able to adhere to the colonic mucosa, thereby reducing the concentration of gut pathogens and modulating the local immunity [95]. Subsequently, in 2014, a randomized double-blind controlled trial assessed the beneficial administration of the probiotics Bifilact (*Lactobacillus acidophilus LAC361* and *Bifidobacterium longum BB536*) on significantly reducing moderate and severe treatment-induced diarrhea during pelvic radiation [96]. On the same line, in 2015, for the first time, a clinical trial evaluated the probiotic formula Colon Dophilus (mixture of 10 different probiotic strains) in the prevention of diarrhea in patients with metastatic CRC, treated with irinotecan-based chemotherapy, suggesting that the administration of such probiotics is safe and leads to a reduction in the incidence and severity of diarrhea and chemotherapy induced gastrointestinal toxicity [97]. In 2016, another double-blind, randomized trial demonstrated that the administration of a combination of prebiotics and probiotics to patients subjected to CRC resection may alleviate irritable bowel syndrome (IBS), often following the operation [98]. In the same year, another trial further analyzed the effects of randomized oral administration of the probiotic *Saccaromices bulardii* in CRC patients. The authors found that this probiotic was able to downregulate pro-inflammatory cytokines in treated patients, although with lacking effects on the post-operative infection rates [99]. Moreover, according to the result of a trial published in 2017, the randomized administration of *Bifidobacterium lactis* and *Lactobacillus acidophilus* to CRC patients, can change the epigenetic patterns of tumor tissue from its baseline, with potential therapeutic benefits in CRC by manipulation of the gut microbiota [100]. The same year a randomized clinical trial with CRC patients demonstrated that the perioperative administration of a mixture of prebiotics and probiotics, significantly reduced postoperative infection rates in patients with CRC [101].

Regardless the observed beneficial effects, larger and controlled clinical trials are further needed to truly endorse both the efficacy and the safety of administering selected species of probiotics during or following anti-cancer treatments (Table 1).

### 5.3. Use of Fecal Microbiota Transplantation (FMT) in Oncology

The exchange of gut microbiota between individuals has been used to cure pathogens infections or in the treatment of gut inflammatory disease and dysbiosis. For example, FMT has been used to cure recurrent *Clostridium difficile* duodenal infection [195,196]. Moreover, FMT has been used in a Graft Versus Host Disease (GVHD) after allogeneic stem cell transplantation [197]. Regarding anti-tumor therapeutic applications, preclinical studies performed in mice demonstrated the efficacy of FMT in reducing colon tumorigenesis, although the efficacy in clinical trials still needs to be further proven [198]. Several clinical trials, designed to evaluate the use of FMT in cancer patients are currently ongoing, with the common goal of preventing and/or ameliorating intestinal side-effects of anti-cancer therapies in cancer patients (Table 1).

Despite the success of FMT, there is still a lack of control in this procedure because the whole gut microbiota is transferred along with the therapeutic bacteria species. Therefore, it is of key importance the careful control of the donors’ health and their gut microbiome specific composition [199].

## 6. Conclusions and Future Perspectives

The relationship between gut resident microbiota and their host is complex. Each individual inherited a specific gut microbiota footprint since their birth, and their intestinal microbiota develops and changes with aging, diet and lifetime exposure to the heterogeneous environment. Indeed, this balance is very delicate and subjected to multiple changes during the entire lifespan.

Nowadays, there is a growing attention towards the characterization of the gastro-intestinal microbiota composition and functionalities. Genetics, together with functional studies, highlighted a dual role played by the gut microbiome in cancer. Some bacterial subpopulations are able to rise during gut dysbiosis and, in turn, to trigger the formation of an inflammatory and pro-cancerogenic environment. On the other hand, many gut derived probiotics are able to protect the host, re-establishing the conditions of a healthy intestinal microbiota within dysbiotic patients, including cancer patients.

LGG is a very good example of a probiotic well studied in cancer, often administered as complementary therapeutic to cure dysbiosis. Given the observed functions as anti-inflammatory and anti-cancer agent in both cellular and animal models, this probiotic may be suggested to be further characterized as adjuvant in integrated anti-cancer therapies in the future.

In line with that observation, this year have witnessed novel breakthrough studies on the association between probiotics and anti-cancer therapy efficacy, as three parallel studies identified specific gut species populating the gastro-intestinal tract of cancer immunotherapy responders, able to improve the efficacy of immunotherapy treatments.

That questions the usage of both probiotics and FMT in cancer therapy, either as tools to repopulate cancer patients’ damaged intestine or even as proper adjuvants in immunotherapy and other kinds of anti-cancer therapies. Correspondingly, care needs to be pursued as patients are often immunocompromised, therefore it is important to evaluate the specific side effects of administering selected bacterial species to such sensitive individuals. In the future, the design of novel experimental trials may undertake a personalized-integrated approach, considering the specific clinical and pathological background of each single patient to be treated, in order to gain only the positive outcomes of probiotics administration and/or fecal transplants, possibly without any harmful side effect.

## Figures and Tables

**Table 1:** Clinical trials * describing the efficacy of probiotics dietary supplementation and/or FMT in cancer patients.

ClinicalTrials.Gov Identifier	Status	Title	Intervention	Disease	Ref.
NCT00936572	C	Probiotics in CRC patients	DS: probiotic *La1*	CRC	[95]
NCT01839721	C	Impact of probiotics on diarrhea in patients treated with pelvic radiation	DS: probiotic *Bifilact*	Various Cancers	[96]
NCT01410955	C	Prevention of irinotecan-induced diarrhea by probiotics	DS: probiotic *Colon Dophilus*	CRC	[97]
NCT01479907	C	Synbiotics and GI function-related quality of life after colectomy for cancer	DS: prebiotics and probiotics *Synbiotic Forte*	CRC	[98]
NCT01609660	C	Impact of probiotics on the intestinal microbiota	DS: *S boulardii*	CRC	[99]
NCT03072641	C	Using probiotics to reactivate tumor-suppressor genes in CRC	DS: probiotic *ProBion Clinica*	CC	[100]
NCT01468779	C	Effect of probiotics in patients undergoing surgery for periampullary neoplasms	DS: probiotics	PC	[101]
NCT01895530	C	Impact of probiotics in modulation of intestinal microbiota	DS: *S boulardii*	CRC	-
NCT03420443	C	Action of synbiotics on irradiated GI mucosa in RC treatment (FIPIREX)	DS: probiotics	RC	-
NCT02771470	C	Intestinal microbiota in lung cancer after chemotherapy	DS: probiotics	LC	-
NCT02021253	C	Influence of probiotics administration before liver resection in liver disease (LIPROCES)	DS: probiotics	HCC	-
NCT02751736	O	The effect of probiotics on bowel function restoration after ileostomy closure in patients with RC	DS: probiotic *CJLP243*	RC	-
NCT03290651	O	Probiotics and breast health	DS: probiotic *RepHresh Pro-B*	BC	-
NCT03518268	O	*Vivomixx* for prevention of bone loss in women with BC treated with an aromatase inhibitor	DS: probiotic *Vivomixx*	BC	-
NCT03177681	O	The effect of yogurt in cancer patient with moderate GI symptoms	DS: probiotics in yogurt	Various Cancers	-
NCT03642548	O	Probiotics combined with chemotherapy for patients with advanced NSCLC	Drug with DS of probiotic *Bifico*	NSCLC	-
NCT03358511	O	Engineering gut microbiome to target BC	DS: Probiotic *Primal Defense Ultra*	BC	-
NCT02944617	O	Probiotic yogurt supplement in reducing diarrhea in patients with metastatic kidney cancer being treated with VEGF-TK inhibitor	DS: probiotics in yogurt	Renal Cell Cancer	-
NCT02351089	O	Probiotics in radiation-treated gynecologic cancer (ProRad)	DS: probiotics	Gynecologic Cancer	-
NCT03574051	O	Microbiota are associated with Iodine-131 therapy and hypothyroidism	Iodine-131 Therapy with DS of probiotics	Thyroid Cancer	-
NCT03552458	O	Effects of Probiotics in preventing oral mucositis in patients undergoing head and neck radiotherapy	DS: probiotic *L Reuteri*	Head-and-neck Cancer	-
NCT02819960	O	prevention of irinotecan-induced diarrhea by probiotics	DS: probiotic *Probio-Fixinum* (including LGG)	CRC	-
NCT01790035	O	Probiotic LGG for prevention of side-effects in patients undergoing chemoradiation for GI cancer	DS: probiotic LGG	GI Cancer	-
NCT00197873	O	*Lactobacillus Rhamnosus* in prevention of chemotherapy-related diarrhea	DS: probiotic LGG	CRC	-
NCT02770326	O	Safety of stool transplant for patients with difficult to treat *C. difficile* infection	FMT	Various Cancers	-
NCT02928523	C	Prevention of dysbiosis complications with autologous FMT in acute myeloid leukemia patients undergoing intensive treatment (ODYSSEE)	Autologous FMT	AML	-
NCT03353402	O	FMT in metastatic melanoma patients who failed immunotherapy	FMT	Melanoma	-
NCT03341143	O	FMT in melanoma patients	FMT with Pembrolizumab	Melanoma	-

* Registered at ClinicalTrials.gov; Abbreviations: C, Closed; O, Ongoing; GI, Gastrointestinal; DS, Dietary Supplement; CRC, Colorectal Cancer; CC, Colon Cancer; RC, Rectal Cancer; BC, Breast Cancer; NSCLC, Non-Small Cell Lung Cancer; PC, Periampullary Carcinoma; LC, Lung Cancer; HCC, Hepatocellular Carcinoma; AML, Acute Myeloid Leukemia.

**Table 2:** The last three-years in vitro and in vivo studies describing the role of LGG in cancer.

LGG-Mediated Effect	Experimental Model	Target Cells	Reference
Anti-inflammatory and anti-cancer effect in colon DMH cancer model	rats	cancer cells	[110]
Dendritic cells exposed to LGG induce TH1 polarization and antitumor response potentiation	ex-vivo immune cells	cancer and immune cells	[111]
Anti-proliferative effects on colon adenocarcinoma cells	cell culture	cancer cells	[102]
Anti-metastatic effects on malignant cells	cell culture	cancer cells	[103]
Promotion of IgA production through upregulation of APRIL expression in intestinal epithelial cells	cell culture and mice	cancer and normal cells	[89]
Change in transcriptome of small intestine cells	mice	normal cells	[112]
Attenuation of the NLRP6-mediated inflammasomes in the intestine	pigs	normal cells	[113]
Prevention of polyp formation in colorectal APC/min cancer model	mice	cancer cells	[108]
Modulation of mTOR and Wnt/β-catenin pathways genes in cancer cell lines (colon, cervical, breast)	cell culture	cancer cells	[107]
Anti-oxidative effects on CC cells	cell culture	cancer cells	[104]
Inhibition of growth of hepatic cancer cells	cell culture	cancer cells	[105]
Anti-cancer effect on oral squamous cell carcinoma	cell culture	cancer cells	[106]
Reduction of colitis associated cancer	mice	cancer cells	[109]
Change of gene expression towards anti-inflammatory profile in intestinal porcine epithelial cells	cell culture	normal cells	[114]
Anti-inflammatory effects in myo-fibroblast colonic cells but not in cancer cells	cell culture and mice	normal cells	[115]
Attenuation of 5-FU-mediated intestinal injury	mice	normal cells	[91]
Protection of gut epithelial cells from radiation injury	mice	normal cells	[92]
Preservation of the gut microbiota balance and intestinal epithelial barrier	pigs	normal cells	[93]
